# EFFECT OF DEPRESSIVE SYMPTOMS AND WORK-RELATED FACTORS ON TRANSITION TO RETIREMENT IN OLDER ADULT

**DOI:** 10.1093/geroni/igad104.3004

**Published:** 2023-12-21

**Authors:** Jennifer Saucedo, Jason Newsom, Em Trubits, Mallory Kroeck, Anda Botoseneanu, Heather Allore, Corey Nagel, Ana Quiñones

**Affiliations:** Portland State University, Portland, Oregon, United States; Portland State University, Portland, Oregon, United States; Portland State University, Portland, Oregon, United States; Portland State University, Portland, Oregon, United States; University of Michigan, Ann Arbor, Michigan, United States; Yale University, New Haven, Connecticut, United States; University of Arkansas for Medical Sciences, Little Rock, Arkansas, United States; Oregon Health & Science University, Portland, Oregon, United States

## Abstract

Work demands can spillover to family domains (work-to-family conflict; WFC) and family demands may spillover to work domains (family-to-work conflict; FWC). This spillover between domains may help explain the relationship between depressive symptoms and decision to retire among older adults. This study examined the influence of depressive symptoms on likelihood of retirement transition through WFC and FWC using data from the Health and Retirement Study (HRS 2008-2018; N = 1031). Participants included adults age 51+ employed in 2008 and reported a transition to partial- or full-retirement by 2018. Cox survival models investigated whether WFC and FWC mediated the effects of depressive symptoms (CESD) on the likelihood of retirement transition, controlling for participant sex, age, education, insurance coverage, and race/ethnicity. Higher depressive symptoms were associated with higher FWC (B = 0.06, p < 0.01) and likelihood of retirement transition (B = 0.07, p < 0.01). Higher FWC was related to lower likelihood of retirement transition (B = -0.89, p < 0.01). The indirect effect of FWC was significant (B = -0.06, p < 0.01). Higher depressive symptoms were associated with higher WFC (B = 0.37, p < 0.01) and likelihood of retirement transition (B = 0.09 p = 0.04). However, there was a nonsignificant effect of WFC on retirement transition (B = -0.19, p = 0.07). WFC had no indirect effect on retirement transition (B = -0.04, p = 0.08). Results suggest that the experiences outside of work for older adults may explain the relationship between depressive symptoms and retirement decisions.

